# Philadelphia County Medical Association

**Published:** 1892-05

**Authors:** G. E. De Schweinitz

**Affiliations:** Professor of Ophthalmology in the Philadelphia Polyclinic; Lecturer on Medical Ophthalmoscopy, University of Pennsylvania; Surgeon to the Philadelphia Hospital, etc.


### Society Proceedings.

### PHILADELPHIA COUNTY MEDICAL SOCIETY.

### SOME CASES OF OBSTRUCTIVE DISEASE OF THE LACHRYMAL PASSAGES AND THE ASSOCIATED INTRA-NASAL LESIONS.

By G. E. De SCHWEINITZ, M. D.,

Professor of Ophthalmology in the Philadelphia Polyclinic; Lecturer on Medical Ophthalmoscopy, University of Pennsylvania; Surgeon to the Philadelphia Hospital, etc.

[*Read March 23, 1892.*]

The intimate relationship between diseases of the lachrymal apparatus—that is, of the drainage system of the eye—and various types of inflammatory changes in the nasal mucous membrane is an old story. Indeed, the close association of ocular and naso-pharyngeal disease is not limited to these conditions. The great majority of phlyctenular ophthalmias depend upon some type of rhinitis, and are often the direct outcome of adenoid growths in the pharynx. Many obscure symptoms which we are wont to describe under the general term asthenopia, have been shown to depend upon intra-nasal disease, and a variety of orbital, ocular, and post-ocular pains are frequently "referred pains;" that is, their origin is from some lesion within the nasal cavity, the frontal sinus, ethmoid cells, or antrum of Highmore. In fact, as Harrison Allen has remarked, a good deal of the success of treatment depends upon a proper attention "to the commonality of the various parts of the cephalic mucous membrane."

The following cases are reported, not because they illustrate new points, but because they emphasize some old ones, and still more because they emphasize that the cure of obstructive lachrymal disease is materially facilitated not merely by the ordinary measures adopted for rendering the passages patent, in association with what may be called routine intra-nasal treatment, (for I take it no one attempts to treat lachrymal disease without due attention to the nasal mucous membrane,) but that more radical measures are frequently of value when applied to the nasal chambers and the vault of the pharynx, which in the vast majority of cases are the regions primarily affected.

Case I. *Purulent dacryocystitis; traces of old rhinitis and abnormal shape of the lower turbinated bone.*—D. D., a boy aged seven years, reported for treatment November 3, 1890. Three years ago pus began to exude from the right punctum lachrymale, and in spite of treatment this condition has continued ever since. The boy was healthy in other respects; he had never suffered from measles nor scarlet fever; was free from the evidences of inherited syphilis, and had sustained no injury. His voice was slightly nasal in tone.

The lower canaliculus was slit, and a firm stricture was found at the beginning of the nasal duct. The probe was not forced; neither was the stricture incised.

The patient was referred to Dr. Alexander MacCoy for nasal examination, who reported as follows: "The right nostril shows an abnormal shape of the lower turbinated bone, also some evidence of a severe rhinitis during the past. I believe that the position and form of the lower turbinated body have had much to do with the disease of the duct on account of the obstruction to its entrance at its lowest portion into the nasal chamber. The boy also has a pharyngeal tonsil, which obstructs the posterior nares somewhat." Dr. MacCoy undertook the treatment of the nasal condition, and after a few days the stricture was incised, the probe passed, and the usual treatment instituted. After the intranasal obstruction was removed, the epiphora ceased and has not reappeared.

I have referred to this case in a paper on the use of pyoktanin in dacryocystitis, (*University Medical Magazine*, Vol. III., p. 181,) and may repeat that my colleague, Dr. Gould, as well as myself, has had favorable effects from this drug in the treatment of unhealthy lachrymal secretions.

The case is now utilized, however, to illustrate what seems to me a very important point to which Dr. MacCoy calls attention in his report, namely, that although the stricture of the duct, which in this case existed high up, was penetrated, and although the fluids and the probe passed readily, the epiphora continued because of the malposition of the turbinated bone. Indeed, this obstruction sometimes exists only in the form of a small flap of mucous membrane, which closes the entrance of the duct into the inferior meatus very much as a valve would do. This effectually prevents the drainage of the eye, and unless it is removed good results will not follow. In this particular instance it was very easy to see the obstruction by first passing a probe and then exposing the entrance of the duct into the meatus by means of a nasal speculum,—a slight precaution which will often lead to the discovery of the cause of a persistent overflow of tears in spite of apparent permeability of the passages.

CASE II. *Catarrhal dacryocystitis; bands of adhesion from the inferior turbinated body to the septum.*—Ella H., aged twenty-eight years, reported for treatment at the Philadelphia Polyclinic, October 24, 1891, on account of an inflammation of the right eye, which had existed for several days. There was a small abscess at the inner margin of the lower lid, with a fistulous communication into the lachrymal sac. A free muco-purulent secretion distended the sac in the form of an ordinary mucocele. The canaliculus had been slit at some previous time, but a probe did not pass readily.

She was referred to the throat department, and examined by Drs. Arthur Watson and Walter Freeman, who reported as follows: "Atrophy of both inferior turbinates; unable to obtain a posterior view; former ulceration of the posterior wall of the pharynx; bands of adhesion from the inferior turbinates to the septum; also one from the middle turbinate to the septum on the right side."

Even in the absence of definite history the pharyngeal condition seemed to indicate syphilis. The patient was ordered an astringent lotion, given potassium iodide and bichloride of mercury, and referred to the throat department for treatment. In January of this year an operation was made upon the lower turbinated bone, and the condition has improved without the passage of probes, the secretion and the epiphora having materially lessened.[1]

This case, it seems, illustrates the ordinary intra-nasal lesions which were evidently at the bottom of the lachrymal trouble, and is further interesting because these lesions gave confirmatory evidence of the syphilitic condition, so much so that relief was facilitated by the proper constitutional remedies.

CASE III. *Lachrymal abscess; spur on the septum opposite the middle turbinated bone; chronic pharyngitis.*—Sarah S., aged forty-five years, reported for treatment at the Philadelphia Polyclinic, November 24, 1891. In April, 1891, epiphora began in the left eye, for which she seems to have undergone no treatment. It continued until about one week ago, when suppuration of the lachrymal sac took place. When she presented herself there was a very marked lachrymal abscess. The pus was evacuated by an external incision, the sac freely irrigated with an antiseptic fluid, and the patient referred to Drs. Watson and Freeman for an examination.

They reported as follows: "On the left side there is a spur on the septum opposite the middle turbinated bone; also hypertrophy of the

1. Recently there has been a relapse in this case Attention to treatment has not been regular.

tissues. The turbinates are small. There is chronic pharyngitis, a thick phlegm covering the tissues."

Unfortunately, this patient has failed to report with any regularity, and the ultimate result cannot be given. This example illustrates the course of so many of these cases, namely, a chronic pharyngitis and hypertrophy and inflammation of the intra-nasal mucous membrane; involvement of the lachrymo-nasal duct; epiphora, owing to an obstruction primarily from swelling of the mucous membrane, and later from the formation of a positive stricture. Under the influence of the pressure and of the stricture, the fluids of the conjunctival sac are not drained, but distending the lachrymal sac, become infective, an abscess forms, and the condition which has been described results.

Case IV. *Epiphora ; atrophic catarrh.*—Jane C., aged sixty years, reported for treatment at the Philadelphia Polyclinic, November 14, 1891, complaining of pain in her eyes, constant epiphora, and inability to read on this account. There was considerable hypermetropia and some astigmatism, and, as epiphora is frequently caused by the strain of uncorrected ametropia, proper glasses were ordered, but the overflow of tears continued. Both canaliculi were then slit. There was narrowing of the ducts, but no stricture, and probe and fluids passed readily. The epiphora improved, but did not disappear.

She was referred to the throat department, and the following report was received : "There is an atrophic condition on both sides, and a spur on the septum on the right side near the opening of the lachrymal duct, but it does not interfere. The closure is probably due to contraction from atrophic changes."

This is a good example of a very common condition, most frequent in elderly people, where there is neither disease of the sac, stricture of the duct, nor pressure from a spur of hypertrophy of the turbinated bodies, but where the obstruction depends upon contraction from atrophic changes.

Case V. *Phlegmonous dacryocystitis ; deflection of the septum ; spur on the left side pressing on the inferior turbinated bone.*—Matthew L., aged twenty-seven years, presented himself for treatment on account of an extensive lachrymal abscess with a small opening and widespread infiltration of the tissues, producing a large swelling involving the lower lid and cheek. The abscess was incised, the pus cavity freely washed out, and an antiseptic dressing applied. In a day or two the swelling had subsided, and nothing remained but a slight browniness of the tissues and a fistulous opening at the point of incision. The canaliculus was slit, but all efforts to introduce the probe proved futile.

The patient had been much exposed to weather; had a history of an old injury, but denied syphilis. The obstruction to the tear passages had existed since the early Fall.

He was referred to the throat department, and the following report was received: "The septum is irregularly deviated in front; there is a spur on the left side pressing on the inferior turbinated body, which also contains an ulcer in its anterior portion."

He was warned that "catching cold," which would increase the nasal obstruction, would certainly bring about a relapse of the abscess. He went to work, however, and returned a few days afterward with all of the lesions previously described in a very much more aggravated state. The same treatment was instituted, and he was again referred to the throat department, and on the 23d of February the hypertrophy on the left side was removed. On the same day a probe was passed, and since this time its passage has been repeated. Epiphora still continues, but is decreasing day by day.

This example illustrates the mechanism of relapse in many of the tear-passage cases, in this instance producing a very serious phlegmonous inflammation. Under treatment and rest sufficient drainage takes place to produce amelioration of the symptoms; then swelling from congestion, owing to exposure, is added to the organic obstruction already present, producing complete closure with an exacerbation such as has been detailed.

CASE VI. *Stricture of the nasal duct; moderate hypertrophy of the inferior turbinated on the left side and a spur on the right side.*—Bridget R., aged fifty years, applied for treatment to the throat department of the Philadelphia Polyclinic, and the following lesions were found: A moderate amount of hypertrophy of the left inferior turbinated near the nasal duct, and a spur on the septum of the right side close to but not obstructing the opening of the duct. With these lesions there were epiphora most marked in O. D., and slight lachrymal conjunctivitis. She had not been able for a number of months to use her eyes with any comfort. She was referred by Drs. Watson and Freeman to the eye department. The canaliculi were slit, and a stricture was found at the mouth of each sac. A No. 2 Bowman's probe was passed without difficulty.

It is evident that although there were lesions in the nasal passages, they were not obstructing the duct, but under the influence of the chronic nasal inflammation a stricture had formed in the lachrymal canal.

CASE VII. *Epiphora from swelling of the mucous membrane of the lachrymo-nasal duct; atrophic rhinitis.*—A. K., an unmarried woman, aged twenty-six years, was referred to me by Dr. Ralph W. Seiss, on account of epiphora of the right eye, which had persisted for some time in spite of the nasal treatment. There was no swelling of the lachrymal sac; no catarrhal or purulent secretion, but simply an overflow of tears. The general health was good, the eyes not far from emmetropic, and there was neither asthenopia nor headache.

Dr. Seiss has kindly furnished the following report of the nasal lesions: "Atrophic rhinitis presenting the ordinary appearances of tissue destruction, combined with some odor and much secondary laryngo-bronchitis."

The canaliculus was slit, and a No. 3 Bowman's probe was passed without meeting a stricture, but with a resistance to its passage, which is characteristic of obstruction from swelling of the mucous membrane. After the passage of this probe the duct was irrigated on several successive days with a solution of boracic acid and common salt, without, however, passing the canula into the duct. The fluid trickled readily through the nose. The epiphora stopped after a few treatments, and has never returned, although many months have gone by since she originally reported.

This patient represents a common class of cases of epiphora associated with chronic inflammation of the naso-pharynx. A somewhat similar inflammation occurs in the nasal duct, but does not produce a true stricture; the occlusion is from swelling, not from cicatricial changes. In many cases it is sufficient to do what was performed in this case; in others even milder measures suffice. Above all things, this is an example of a class of cases the successful treatment of which I have learned especially from Dr. Risley, by obeying the principle which he was wont to instil, not to be too ready to pass probes and canulas, lest their introduction scrape away some of the mucous membrane, and really do more harm than good. It is unnecessary to do more than medicate the swollen mucous membrane with any solution that is suitable; I like boracic acid and common salt very much.

Many more cases might be quoted, but these seven representatives of various classes are sufficient to illustrate the points which I desire to make:

1. A large class of cases exists characterized chiefly by epiphora without catarrhal or purulent secretion, in which the obstruction in the lachrymo-nasal duct depends upon swelling of its mucous membrane, and not upon true stricture. The primary origin of these cases in the great majority of instances is a chronic or subacute post-nasal catarrh. The evident indication is the treatment of the latter condition and the medication of the swollen mucous membrane of the lachrymo-nasal duct, so that it may regain, as nearly as possible, its natural condition, which it will do without much instrumental interference,—an interference that may of itself, if unskilfully performed, be the cause of a cicatrizing band that never originally existed. Case VII. of the series illustrates this class.

2. The life history, if I may so express myself, of many cases of obstructive disease of the lachrymo-nasal duct and the formation of a lachrymal abscess is illustrated by Cases III. and VI. First, a chronic pharyngitis occurs; later, hypertrophy and inflammation of the intranasal mucous membrane, followed by swelling of the lining tissue of the lachrymal duct. Gradually cicatricial changes arise, and a true stricture is formed. The drainage of the conjunctival cul-de-sac ceases; the micrococci natural to the part, and those which readily find access to this region, permeate the contents of the lachrymal sac, because this can no longer be emptied; the pathogenic microörganisms exercise their true function, and suppuration occurs.

3. A number of cases develop, chiefly in old people, in which there is epiphora, again without the presence of pus or muco-pus, depending upon obstruction in the lachrymal duct from atrophic changes, the whole being part of a similar atrophic process in the intranasal passages, and generally described under the term atrophic catarrh. The obstruction in these instances is not from swelling, nor from stricture, but from contraction. Case IV. of the series is an example.

4. A very common cause of an exacerbation of lachrymal disease is due to the pressure of a hypertrophic turbinated body, or similar intranasal obstruction, which, under treatment, has gradually subsided, but which, owing to exposure, swells up again, and exercises its obstructing influence. At once there is occlusion of the lachrymal passages and recrudescence of the symptoms. The very serious nature of such cases is illustrated in Case V. of the series.

5. In every case of local disease the physician should be mindful of constitutional causes; the value of confirmatory evidence by pharyngeal and intranasal examination is illustrated in Case II., an example of constitutional syphilis. Local treatment may be very necessary; local treatment without general medication is ineffectual.

6. Finally, I come to the class of cases in which there exist an obstruction in the intranasal end of the duct (it may be trivial), permeable by the fluids used in a syringe, but an impassable barrier to the outflow of tears. Even the slightest obstructions, under these circumstances, may defeat the most classical treatment of lachrymal disease. The ready detection of such a lesion is illustrated in Case I. of the series.

It has not been my intention this evening to refer to what are the best means of treating lachrymal disease, except in so far as these are implied by the descriptions of the lesions which existed in the examples I have reported. Whether we believe that small or large probes should be passed ; whether we class ourselves with those who believe that the probes should not be used at all; whether we are the advocates of this or that antiseptic and astringent fluid ; whether we think that strictures should be incised or should not be incised, or whether we believe in the permanent wearing of styles or canulas, it is evident that the rational treatment of certain types of obstructive lachrymo-nasal disease must also include not alone the ordinary intranasal treatment with sprays and powders, but a systematic and thorough examination of the naso-pharynx, and, if necessary, the best operative interference known in intranasal surgery.

### DISCUSSION.

Dr. Edward Jackson : I have very little to add to this paper. We are not in a position to generalize widely on this subject, or determine how many cases, or what proportion of cases, belong to that group in which the obstruction comes originally from the nasal chambers. Certainly I have not studied enough of these cases to go further than to simply consider individual instances and study the lessons that they seem to teach. A case that comes now to mind is one that was treated some years ago at the Polyclinic for lachrymal obstruction. He recovered, or at least got into such a good condition that he ceased to attend. Within a few months he returned to the clinic with a renewal of his epiphora, and on passing a probe I found no obstruction until the lower end of the duct was reached. There the obstruction was very noticeable, although no great difficulty was experienced in passing the probe. He was referred to the nose and throat department, and there was found a cicatrix involving the lower end of the duct. Whether this cicatrix was connected with the former treatment, or whether it resulted from the original nasal lesion, I am not prepared to say. Its excision certainly removed the obstruction to the flow of tears.

I recall two cases in which the thickening of the mucous membrane at the opening of the duct into the nose was the sole cause of the epiphora. Probably in the great majority of cases of lachrymal obstruction the original obstruction has been seated at one end of the canal. I do not think that it is always, and perhaps not in the majority of cases, that the trouble begins at the lower end of the canal coming from the nasal chambers. Frequently it commences with the puncta. Some of the obstinate cases that have come to me with a history of slitting up of the canaliculi, and long continued treatment with probes without permanent benefit, have shown a grave error in the position of the incision into the canaliculus. Instead of the conjunctival surface, the cut has been made on the upper edge of the lid, so that the tears could not get into the passage until they ran over the edge of the lid. These cases are liable to a return of the acute trouble, for if the normal flow of the fluid through the lachrymal sac and duct is not sustained, micrococci which enter find the conditions most favorable for free development and the setting up of pathological processes.

Dr. SAMUEL D. RISLEY: The facts which Dr. de Schweinitz has set forth in this admirably reported group of cases are of great practical importance, both to the ophthalmologist and those who treat the diseases of the nas-opharynx. The conditions so aptly described suggest many points of great importance. It recalls some of my early experiences in the treatment of lachrymal disease. I remember the case of a Mr. C., whom I had treated for a long time in 1879 for lachrymal retention unsuccessfully. There was no stricture of the duct other than that due to a more or less uniform thickening of the mucous membrane, but there was, nevertheless, more or less constant epiphora. Incidentally, he called my attention to some trouble with the nostril on the side of the affected tear duct. I discovered a broad superficial ulcer underlying the anterior end of the inferior turbinated bone. This was speedily cured by a few applications, and his lachrymal trouble soon disappeared. This was the first inkling I had received of the important relation which might exist between certain cases of lachrymal disease and affections of the nasal passages. At that time, so far as I knew, literature was silent upon the subject. From that time to this it has been my uniform practice to carefully inspect the nose in every case of lachrymal disease.

Dr. de Schweinitz's paper is an admirable statement of facts, with which my own experience is strictly in accord in a large group of cases suffering from this very troublesome and persistent affection. These facts explain why so many cases of epiphora present no marked stricture of the lachrymal duct. I have also had experiences the counterpart of that related by Dr. Jackson, where the probe was passed freely until the nasal end of the duct was reached, and there, meeting with resistance, if forced roughly into the nose will cause bleeding from laceration of the inflamed and swollen mucous membrane, closing or blocking the nasal orifice of the canal.

Another practical bearing of these facts in ophthalmic surgery is, that since the lachrymal passages are liable to disease by extension upward from the nose, which furnishes such perfect conditions for the rapid development of microörganisms, the nasal passages may become the source of infection for the eye itself. It suggests the necessity for great care in this direction, particularly before and after operations upon the eye. We may deluge the conjunctival sac with antiseptic lotions before opening the anterior chamber, bandage the eye and imagine that all has been done for the safety of our patient, whereas the facts set forth this evening suggest the possibility of infection from the nose through the lachrymal duct. With this possibility in mind, I have of late years recognized the importance of washing out the lachrymal sac and nasal passages with bichloride of mercury solution where I expect to bandage the eyes after operation.

If affections of the nasal mucous membrane are then the origin of a considerable group of cases of lachrymal disease, it is obviously unwise to treat the duct harshly by probing until after the nasal disease is excluded. The function of the lachrymal duct is not performed in the manner of a drain-pipe, but is rather a capillary tube, and its inflammations may often be cured by washing with suitable lotions. Probing is often necessary, but rarely with the idea of dilating the capillary tube into an open canal. In 1877 I urged that the proper function of the probe was to induce absorption of the products of inflammation in the thickened membrane lining the duct, rather than the rupture of a stricture or dilatation of the duct.

Dr. Alexander B. Randall: I do not think I can add anything to what has been said. I have not met with a great deal of lachrymal trouble in the three or four thousand cases seen at the Episcopal Hospital. I have met, in that number, with only thirty that required absolutely lachrymal treatment. I recall a large number of cases where the nasal trouble seemed to be the cause of the affection, and where treatment directed solely to the nose has resulted most happily. In a large number of cases of children with watery eyes I have never, I believe, with one exception, used any other treatment than that to the nares and lower end of the duct, and have had no reason to regret the absence of other forms of treatment. I have always thought that the puncta, the arrangement of the canaliculi, and the arrangements of the upper part of the lachrymal passage, had a decided physiological purpose, and that it was a great disadvantage to treat these parts by incision and probing if there were no absolute necessity for it. In directing attention to the primary incision and to the constitutional treatment of the case, my results have been most satisfactory with a minimum amount of necessity for surgical procedures directed to the upper part of the lachrymal passages.

Dr. Ralph W. Seiss: With regard to the nasal lesions found in these cases, in the instances that I have seen they have been almost altogether of two types. One is enlargement of the anterior nasal spine with ecchondroses of the septum and swelling of the mucous membrane; the other is atrophic and sclerotic changes. It is important to keep this in mind, as it has an important bearing upon the treatment. The galvano-cautery is an admirable agent in the treatment of nasal troubles, but it must be used with caution in these cases. I have seen seven or eight cases of lachrymal obstruction following the reckless use of this agent to the lower turbinated body. When I receive such a case for treatment I am more apt to use trichlor-acetic acid or a single crystal of chronic acid than the cautery.

Dr. L. Webster Fox: There was one point which was not discussed by Dr. de Schweinitz in his paper, and yet my observations have led me to believe that it plays a very important rôle in the causation of lachrymal disturbances, and that is, the asymmetry of the face. A deviation from the middle line by the nasal bones or septum would perforce cause a modification of the calibre of the lachrymal canal on that side. Any irritating substance lodged in this constricted channel could not find easy escape, and in consequence inflammation develops which evidently would lead on to lachrymal abscess. Then, again, closure of both upper and lower openings of the canaliculi, caused by chronic conjunctivitis or blepharitis, proves again that asymmetry must play a factor in these cases, for with both eyes afflicted more or less, but one side of the drainage canal is affected. In 1884 all cases of lachrymal obstruction applying to the eye department of the Germantown Hospital were referred to Dr. S. MacSmith for nasal examination. I was in hopes that we could trace all lachrymal disorders to disturbances in the nasal cavities, but we were doomed to disappointment. While a certain number of cases had undoubted nasal complications, yet in many the lesion was found on the side opposite to the epiphora or lachrymal abscess. In some few cases we found the applied treatment to the nasal cavity did not give relief, but in the majority we found that you must apply treatment to the orbital end of the canal to obtain good results. Dr. de Schweinitz did not dwell upon the treatment of lachrymal disturbances, which I regret; for, after all, we desire to learn from each other the best means by which a cure may be brought about, or at least to alleviate our patients. My experience has led me to adopt the larger Cawper probes, followed by the insertion of a silver tube. In certain forms of epiphora a simple dilatation of the mouths of canaliculi will alleviate the patient, or slitting up, as suggested by Mr. Bowman; but where you have a stricture or lachrymal abscess, or both, I adopt the radical treatment – dilatation to its fullest capacity. As regards the application of astringent washes, I have never had much success from their use alone.

Dr. Charles Hermon Thomas: The paper which Dr. de Schweinitz has read is an interesting and valuable contribution to the treatment of lachrymal obstruction. It, and especially the discussion which has taken place, has strongly emphasized a phase of the subject on which I confess I have not laid much stress in my own experience, which has withal been a not unsuccessful one. For a good many years I have had such satisfaction in the treatment of these cases as to leave little to be desired. I do not doubt that many of these cases can be relieved from the nasal side, but I must believe that there are a number of cases which can hardly be treated successfully in this way, exclusively cases in which the irritation has been so long continued that it has resulted in what might be called organic stricture as distinct as stricture of the urethra. Such cases certainly demand local treatment at the point of obstruction.

It was in 1868 that Stilling made the announcement of the results obtained by the use of the knife which he devised for the purpose of cutting strictures of the lachrymal duct. I was impressed with the value of the method of treatment proposed by him, and also with the want of adaptation of the knife which Stilling figured for the purpose. It seemed to me that the stiff, conical blade was faulty. I, therefore, devised a knife with a blunt conical tip, with the edge so set as to cut in withdrawing only, and attached to a flexible shank so that it could be bent to conform to the shape of the bony canal, and yet be rigid enough to control the blade. By slitting the lower canaliculus and first passing some of the more delicate probes, especially those of Dr. Williams, of Boston, this knife may be slipped down and a free lineal incision made. The stricture is then divided completely even to the bone, and a large leaden style is introduced and allowed to remain for a few days or weeks at most, being removed daily for a time, for the purpose of washing the passage with some antiseptic fluid. By this method I have had such success as seems to leave little to be desired, and can hardly think that the time has arrived to abandon that method altogether and turn these cases over to the rhinologist. Indeed, I do not now recall a single case in which I had difficulty from obstruction at the lower end after I had gotton a passage through. The facts brought out by Dr. de Schweinitz doubtless make it most desirable to have the nasal passages of patients suffering from epiphora examined, and any abnormalities found therein treated. It is my purpose to return to this subject in the near future, and to enter more into details as to the method of treatment here briefly sketched.

Dr. George M. Gould: I wish to go one step further than Dr.

Thomas in emphasizing the importance of ophthalmological treatment as such. There can be no question as regards cases such as Dr. de Schweinitz has presented. When there is absolute impermeability of the nasal end of the duct, the treatment is, of course, outlined by the diagnosis. In the greater number of cases, however, there is not absolute obstruction of the duct, but simply a stenosis, an unhealthy congested condition of the lachrymal mucous membrane, the duct certainly being patent to some extent, but not enough to carry off the large excess of tears. The frequent use of probes has seemed to me not only not necessary but simply superfluous in these cases.

During the past year I have employed a plan of treatment in such cases, which has been so successful that I shall outline it. It consists in slitting the punctum vertically downward toward the palpebral fold, in order to increase the size of the opening. Then canting the patient's head to one side, the corner of the eye is filled with an antiseptic astringent lotion. The duct should first be emptied by pressure, and then allowed to fill with this solution. This procedure of emptying and refilling the duct is repeated several times, and thus the antiseptic solution is brought in contact with canaliculus, sac, and duct by capillarity and pressure. I have had cases in which, after showing the patient the method once, he has afterward practised it himself and came back in a week perfectly cured. The method is simple and effective, and can be carried out at home. I have often wondered in those cases where probing has been employed, whether it was the probe or the antiseptic lotion that had done the good.

In regard to slitting of the canaliculus, I may say that I do not do this at the beginning of the treatment. If there is narrowing of the puncta, the fluid enters more readily if it is cut.

Dr. George Friebis: I should like to ask whether, in the experience of Dr. de Schweinitz, he has met with obstruction due to such causes as inflammation and enlargement of the caruncle? I have in mind one case (a male adult, past middle age,) in which I paid little attention to the inflamed caruncle, and the case did not improve under the routine treatment. Upon recognizing the inflammation of the caruncle as a possible cause, and treating it with astringents, I succeeded in curing the epiphora without further instrumental interference.

Dr. de Schweinitz: I have presented this series of cases simply for the purpose of classifying one of the many varieties of lachrymal obstruction. I beg Dr. Thomas will not think that I wish to transfer the treatment of lachrymal obstruction to my friends, the rhinologists, much as I value their aid in the management of some of these cases, and I heartily agree with Dr. Thomas and with Dr. Gould, that the ophthalmological treatment of lachrymal obstruction is of paramount importance. These cases illustrate merely certain failures in treatment when applied to the ducts alone, because there is obstruction either at the inferior end of the duct or from intranasal lesion. I have not intended this evening to include the large number of cases due to obstruction in the canaliculus from polypi, from tear-stones, from fungus, or to the cases of obstruction high up, or to those which result from conjunctivitis and from malposition of punctum lachrymale. Dr. Fox's observation in regard to a symmetry of the face is an important one, and deserving of much study. In regard to the use of large probes, I might not find myself in accord with Dr. Fox. Abnormal position of the caruncle or its enlargement, as referred to by Dr. Friebis, is an interesting anomaly. You are all familiar with the cases reported by Von Graefe and by Horner. I have some knowledge of a similar case occurring in the practice of Dr. Wallace, of this city. Cases of this character, or others which have been brought up into discussion, have been purposely omitted in the paper of this evening. My idea was simply to show that certain examples exist, and they are not infrequent, which can be treated better with than without the aid of the rhinologist.

